# Identification and Expression Analysis of Zebrafish (*Danio rerio*) E-Selectin during Embryonic Development

**DOI:** 10.3390/molecules201018539

**Published:** 2015-10-12

**Authors:** Guijin Sun, Kechun Liu, Xue Wang, Xiuhe Liu, Qiuxia He, Chung-Der Hsiao

**Affiliations:** 1 Biology Institute, Shandong Academy of Sciences, Jinan 250014, China; E-Mails: xuewang1978@126.com (X.W.); qiuxiahe1980@126.com (Q.H.); 2 School of Food Science and Engineering, Qilu University of Technology, Jinan 250014, China; E-Mails: sgjsyh@163.com (G.S.); sbchen2007@163.com (X.L.); 3 Department of Bioscience Technology, Chung Yuan Christian University, Chung-Li 32023, Taiwan; E-Mail: cdhsiao@cycu.edu.tw

**Keywords:** zebrafish, E-selectin, identification, expression, lipopolysaccharide

## Abstract

In this study, we cloned the full-length cDNA of E-selectin of zebrafish (*Danio rerio*), analyzed its expression pattern and preliminarily explored its biological function. Zebrafish E-selectin cDNA is 3146 bp and encodes a putative 871 amino acid protein. All structural domains involved in E-selectin function are conserved in the putative protein. Whole-mount *in situ* hybridization of zebrafish at 24 and 48 h post-fertilization (hpf) revealed E-selectin expression mainly in vascular/endothelial progenitor cells in the posterior trunk and blood cells in the intermediate cell mass and posterior cardinal vein regions. Real-time quantitative RT-PCR analysis detected E-selectin expression at 0.2, 24 and 48 hpf and significantly decreased from 48 to 72 hpf. The expression of E-selectin, tumor necrosis factor-α and interleukin-1β was significantly upregulated at 22 to 72 h after induction with bacterial lipopolysaccharide. Thus, the structure of E-selectin protein is highly conserved among species, and E-selectin may be involved in embryonic development and essential for hematopoiesis and angiogenesis during embryonic development in zebrafish. Furthermore, we provide the first evidence of inflammatory mediators inducing E-selectin expression in non-mammalian vertebrates, which suggests that zebrafish E-selectin may be involved in inflammation and probably has similar biological function to mammalian E-selectin.

## 1. Introduction

The three known selectins, L-, E- and P-selectins, constitute a family of calcium-dependent cell surface glycoproteins that play a critical role in inflammation, mainly via recognition of specific carbohydrate ligands, sialyl Lewis X (sLe^X^) and sialyl Lewis A (sLe^A^) [1,2]. Structural features common to the selectins are the presence of five distinct structural domains involved in their function: an N-terminal C-type (Ca^2+^-dependent) lectin binding domain, an epidermal growth factor (EGF)-like domain, a variable number of short consensus repeat (SCR) units similar to those in complement-regulatory proteins, a transmembrane domain, and a short cytoplasmic domain [3,4]. The selectins are involved in a wide array of interactions between leukocytes and endothelial cells [5]. E-selectin is believed to mediate the initial adhesion of leucocytes to the endothelium and support the adhesion of a number of different types of leucocytes, including granulocytes, monocytes and particular lymphocyte subsets, to the endothelium [1,3,4,6].

E-selectin is not constitutively expressed in endothelial cells (ECs), but its expression is induced by tumor necrosis factor-α (TNF-α), interleukin-1β (IL-1β), IL-4 and bacterial lipopolysaccharide (LPS) [7,8]. E-selectin and its ligands are believed to play important roles in many types of inflammatory diseases, including atherosclerosis, psoriasis, diabetes, dermatitis, asthma and cancer [1,9,10,11,12,13,14]. In addition to E-selectin-mediated inflammation, many studies have suggested a potential involvement of E-selectin in the attachment and transmigration of circulatory metastatic cancer cells through the endothelium [15,16]. Consequently, E-selectin has been highlighted as a potential therapeutic target [4,5].

Zebrafish (*Danio rerio*) has become a prominent vertebrate animal model for investigating human diseases [17,18,19,20] because of its high fecundity, external embryonic development, transparency of the embryos, advanced genomic resources, and similar organ systems and gene sequences as in humans. To our knowledge, E-selectin is largely uncharacterized in zebrafish. In this study, we cloned the full-length cDNA of zebrafish E-selectin and analyzed its expression pattern for possible use of E-selectin as a therapeutic target and better use of zebrafish as a model organism for human disease. We preliminarily explored its biological function by induction with LPS.

## 2. Results and Discussion

### 2.1. Cloning of Zebrafish E-Selectin

E-selectin was first identified as a cytokine-inducible surface glycoprotein involved in the binding of leukocytes to cultured human umbilical vein endothelial cells [6]. Subsequently, the E-selectin gene was isolated and characterized in rabbit [21], mouse [22], rat [23], cow [24], pig [7] and horse [25] models. In this study, we characterized zebrafish E-selectin to facilitate future studies of inflammation and EC activation in zebrafish. To our knowledge, this cloning of zebrafish E-selectin represents the first in non-mammalian vertebrates. 

### 2.2. Nucleotide Sequence Analysis of Zebrafish E-Selectin

Zebrafish E-selectin cDNA is 3146 bp and contains a 102-bp 5′-UTR, a 2613-bp coding sequence and a 431-bp 3′-UTR. The sequence was deposited in GenBank (accession no. KC488324). Zebrafish E-selectin gene contains 17 exons and 16 introns and is located on chromosome 20.

### 2.3. Amino Acid Sequence Analysis of Zebrafish E-Selectin

**Figure 1:**
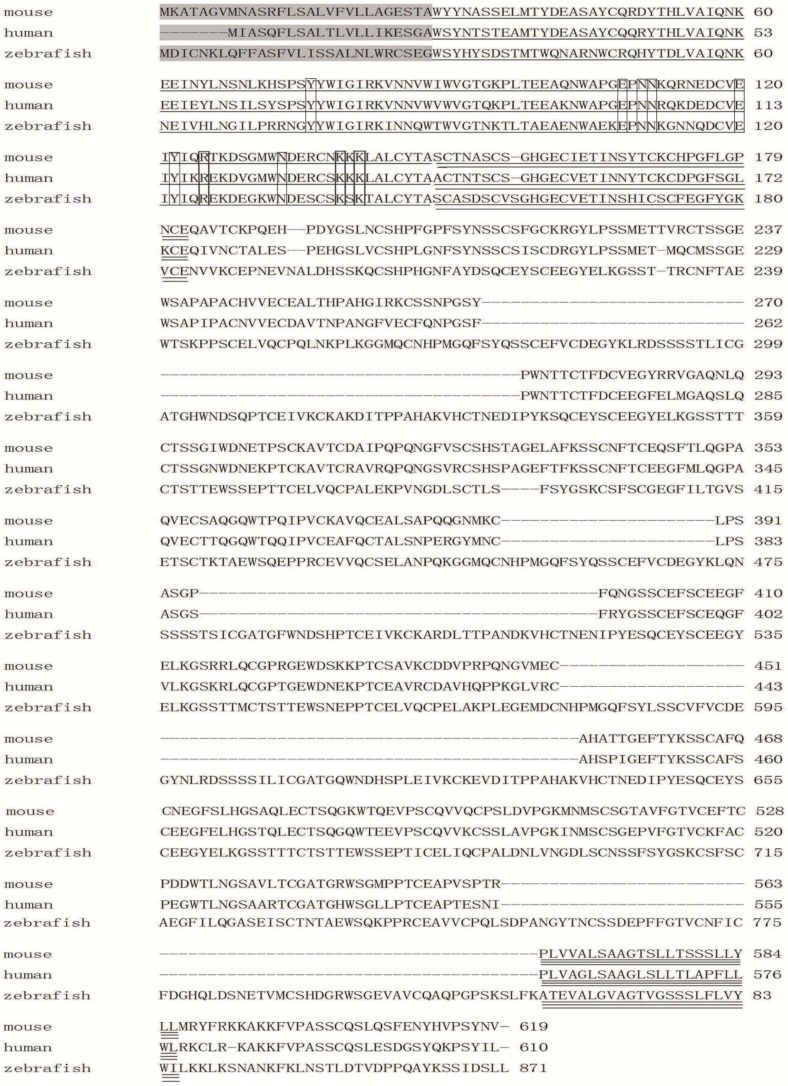
Comparison of mouse, human and zebrafish E-selectin protein sequences. The signal sequence of each protein is shaded. The lectin domain is single-underlined, the EGF-like domain is double-underlined, and the transmembrane domain is triple-underlined. The SCR domain is between EGF-like and transmembrane domains, and the cytoplasmic tail is behind the transmembrane domain. The amino acid residues predicted to be important for neutrophil adhesion and in calcium binding are boxed.

Zebrafish E-selectin cDNA encodes a putative 871 amino acid protein, with the first 28 amino acids probably serving as a signal peptide because this region closely matches the human signal sequence (Figure 1). The mature protein was predicted to consist of a 787 amino acid extracellular region, a 23 amino acid transmembrane region and a 33 amino acid cytoplasmic tail (Figure 1), with a theoretical molecular weight of 92.95 kDa and seven potential N-linked glycosylation sites.

**Figure 2:**
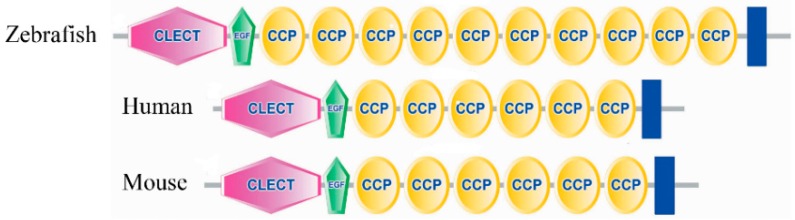
The conserved structural domains of zebrafish, human and mouse E-selectin by SMART analysis.

The amino acid sequence of zebrafish E-selectin is 40.71% to 43.44% identical to that of mammalian E-selectins. Moreover, five structural domains putatively involved in E-selectin function are conserved in the putative zebrafish protein and include a lectin domain, an EGF-like domain, a 10-SCR domain, a transmembrane domain and cytoplasmic tail (Figure 1 and Figure 2). 

Despite low amino acid sequence homology between zebrafish and mammals, five distinct structural domains involved in E-selectin function are conserved. The amino acid residues (tyr76, asn110, asn111, glu120, tyr122, arg125, lys139 and lys141) predicted to be important for neutrophil adhesion to human E-selectin are all conserved in the zebrafish amino acid sequence [26] (Figure 1). The residues glu108, asn110 and asn133, believed to be important in calcium binding, are also conserved [26] (Figure 1). Thus, the primary structure of E-selectin protein is relatively conserved across species, which strongly suggests that the conserved structural domains may be the basis of the consistent E-selectin function in all vertebrates.

Alignment of E-selectin between zebrafish and mammals indicates the amino acid sequences of the lectin and EGF-like domains are relatively highly conserved (57.03%–61.72% and 50%–64.71%, respectively), with greater conservation than the SCR, transmembrane and cytoplasmic domain sequences (34.44%–39.95%, 4.35%–39.13% and 12.12%–25%, respectively). The relatively high conservation of the lectin and EGF-like domains suggests that these two regions are important for E-selectin function and supports that the lectin and EGF-like domains are directly involved in cell adhesion and may determine the specificity of ligand binding [27]. 

Among the five domains, the number of SCR domain varies among species. Zebrafish contains 10 SCR domains; human, mouse and horse, six; rat and rabbit, five; and cow, pig, sheep and deer, five. The putative protein is 252 to 261 amino acids longer than that for mouse (GenBank accession no. AAA37577), human (accession no. NP_000441) and horse (accession no. NP_001075324); 320 to 322 amino acids longer than that for rabbit (accession no. NP_001075781) and rat (accession no. NP_620234); and 386 to 389 amino acids longer than that for cow (accession no. AAA02991), deer (accession no. AAK48710), sheep (accession no. NP_001009749) and pig (accession no. NP_999433). 

A SCR domain contains approximately 60 amino acids and the lengths in E-selectin proteins vary, mainly because of differences in number of SCR domains among species. The function of the SCR domain remains undetermined. McEver demonstrated that it simply acted as a structural scaffold to facilitate the efficient interaction of the lectin and EGF-like domains with their counter-receptor [28]. The variable number of SCR domains in these species suggest that the domain is not as critical as the lectin and EGF-like domains in the proper functioning of the molecule, which supports McEver’s theory.

The function(s) of the five conserved domains in ligand binding and cell adhesion has been a major study focus. Characterization of zebrafish E-selectin may provide insight into understanding the functions of these conserved domains.

**Figure 3:**
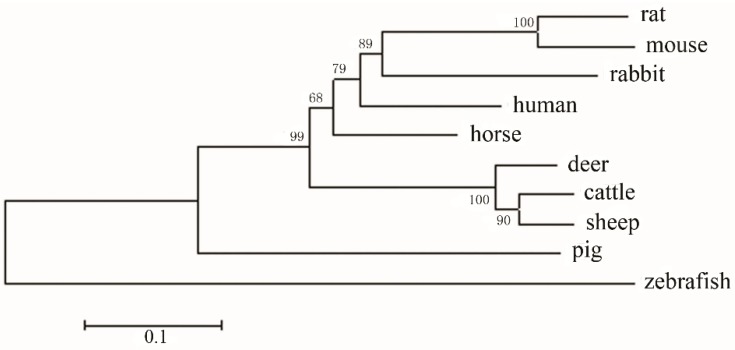
Phylogenetic analysis of E-selectin family members. The numbers indicate the bootstrap confidence values for each node with 1000 replications, for human (GenBank accession no. NP_000441); mouse (no. AAA37577); sheep (no. NP_001009749); horse (no. NP_001075324); cow (no. AAA02991); rat (no. NP_620234); rabbit (no. NP_001075781); pig (no. NP_999433); and deer (no. AAK48710).

To study the evolutionary relationship of zebrafish and mammalian E-selectin, we constructed a phylogenetic tree by aligning amino acid sequences. The 10 species were clearly divided into two branches: mammalian species and zebrafish (Figure 3). Mammalian and zebrafish E-selectin belonged to distinct clades, which indicates a relatively distant evolutionary relationship between mammals and zebrafish and is consistent with the biological classification.

### 2.4. Expression Pattern of E-Selectin during Zebrafish Embryonic Development 

To clarify the role of zebrafish E-selectin, we analyzed its spatial and temporal expression patterns during embryonic development by real-time quantitative RT-PCR and whole-mount *in situ* hybridization. 

**Figure 4:**
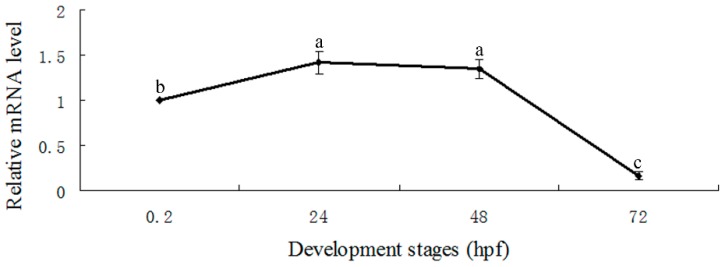
Real-time quantitative RT-PCR analysis of expression of E-selectin during zebrafish embryonic development. Data are mean ± S.D. Means with different letters (a, b, and c) indicate significant difference (*p* < 0.05).

**Figure 5:**
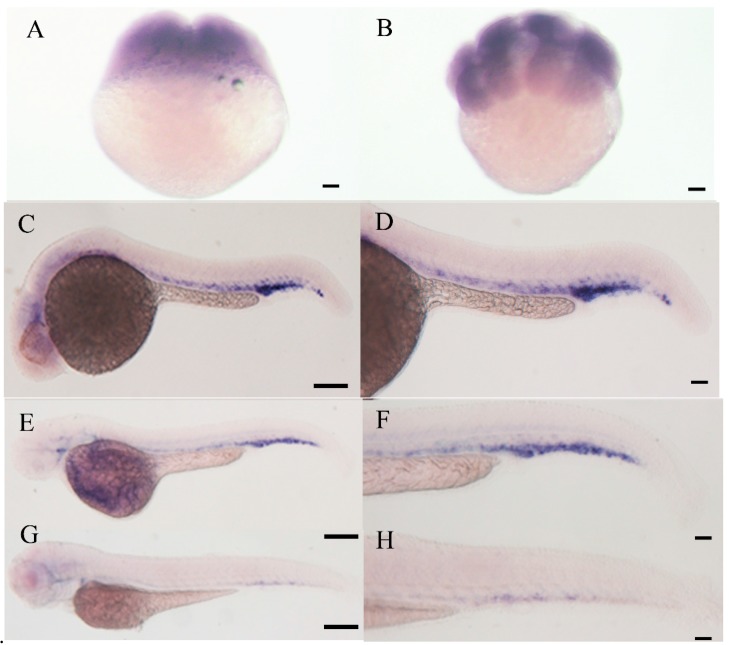
Whole-mount *in situ* hybridization analysis of expression of E-selectin during zebrafish embryonic development. (**A**) 2-cell stage, (**B**) 8-cell stage, (**C**, **D**) 24 hpf, (**E**, **F**) 48 hpf, (**G**, **H**) 72 hpf. (**D**, **F**, **H**) Enlarged view of trunk and caudal region. Scale bars, 5 μm.

Real-time quantitative RT-PCR revealed E-selectin expression at 0.2, 24 and 48 h post-fertilization (hpf) and significantly decreased from 48 to 72 hpf (Figure 4). Whole-mount *in situ* hybridization detected E-selectin expression at 2- and 8-cell stages (Figure 5A,B). The expression was observed in vascular/endothelial progenitor cells in the posterior trunk and blood cells in the intermediate cell mass and posterior cardinal vein regions at 24 hpf (Figure 5C,D) and was weaker in these areas at 48 hpf (Figure 5E,F). The expression decreased significantly at 72 hpf (Figure 5G,H).

These results suggest that E-selectin is expressed maternally and plays an important role in embryonic development, particularly in hematopoiesis and angiogenesis. To our knowledge, the expression pattern of E-selectin has never been reported in other species, so our examination of its expression in zebrafish provides some clues to its function in embryonic development. 

### 2.5. LPS-Induced Expression of E-Selectin, TNF-α and IL-1β

**Figure 6:**
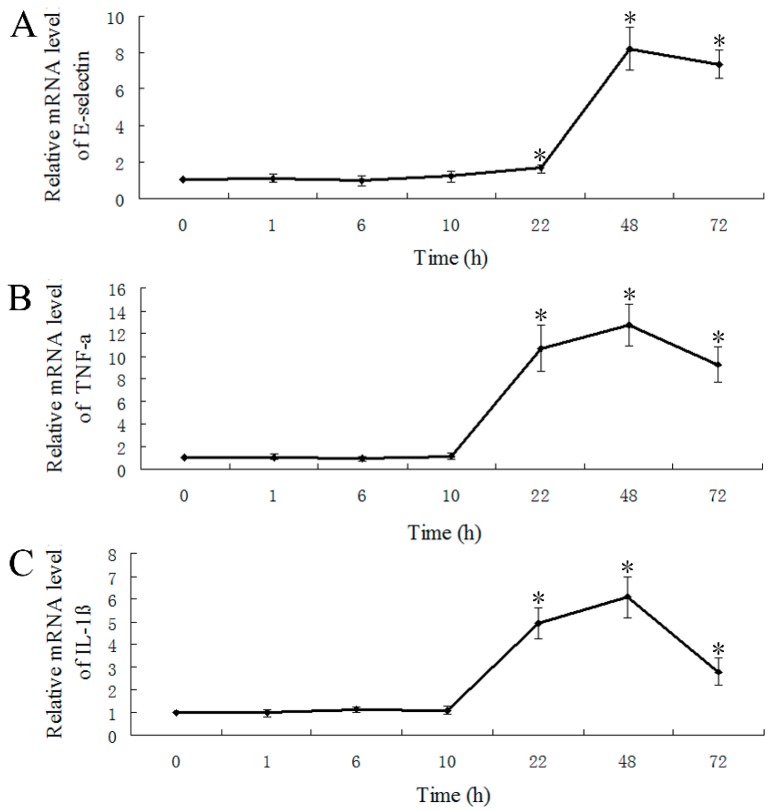
Real-time quantitative RT-PCR analysis of mRNA levels of (**A**) E-selectin, (**B**) TNF-α and (**C**) IL-1β with lipopolysaccharide induction. Data are mean ± S.D. * *p* < 0.05 compared with control (0 h).

E-selectin is not constitutively expressed in ECs. *In vitro* and *in vivo* studies have shown that the inflammatory mediators induce the expression of E-selectin mRNA and protein in mammals [7,8,29]. In the inflammatory response, the pro-inflammatory cytokines IL-1β and TNF-α are secreted and are primary regulators [30,31,32]. The two cytokines play a critical role in initiating the pro-inflammatory cytokine cascade, in recruitment and activation of macrophages, and in stimulation of the adaptive immune response [31]. To further investigate the biological function of zebrafish E-selectin, we analyzed the expression of E-selectin, TNF-α and IL-1β by LPS induction over 72 h by real-time quantitative RT-PCR. The mRNA levels of E-selectin, TNF-α and IL-1β were significantly increased at 22 h and peaked at 48 h, then decreased at 72 h (Figure 6).

We show the induction of E-selectin expression in non-mammalian vertebrates by an inflammatory mediator, LPS. Zebrafish E-selectin may be involved in inflammation, similar or identical to mammalian E-selectin. Previously, E-selectin protein synthesis was detected in stimulated ECs [7,25]. In the present study, we detected mRNA expression. Further investigation of zebrafish E-selectin protein expression with LPS induction is required.

## 3. Experimental Section 

### 3.1. Cloning of Zebrafish E-Selectin cDNA 

We performed a BLAST search of the NCBI database (http://www.ncbi.nlm.nih.gov) and obtained the predicted zebrafish E-selectin cDNA sequence (GenBank accession nos. XM_005160715 and NM_001130598) and corresponding protein sequence (accession nos. XP_005160772 and NP_001124070). We used the predicted cDNA sequence to design primers (5′-AAGTCCACTGCA CTAATGAA-3′ and 5′-GTTGAAGTGCACGTCGTTGT-3′) to amplify partial cDNA for zebrafish E-selectin by RT-PCR. RT-PCR involved use of total RNA extracted from embryos at 24, 48 and 72 hpf; amplification conditions were 3 min at 94 °C; 35 cycles of 94 °C for 30 s, 55 °C for 30 s, 72 °C for 1 min and a final extension at 72 °C for 10 min. We used the cloned cDNA fragment sequence to design the primer sequences (5′-ACAGCGAGGTGGTTCCTGACTCCACTCT-3′ and 5′-CCACTGGAAGG TGAAATGGATTG-3′) to amplify 5'- and 3'-untranslated regions (UTRs) with 5′-rapid amplification of cDNA ends (5′-RACE) and 3′-RACE with the SMART-RACE cDNA Amplification Kit (Clontech, Mountain View, CA, USA). We used PCR with the primer sequences (5′-CTCCCAATCAAAGCCATCAAAC-3′ and 5′-TTGGAGTTATTTCAGTGACTTCAAC-3′) to amplify the full-length cDNA of zebrafish E-selectin for verification. The amplification program was 94 °C for 5 min; 35 cycles of 94 °C for 30 s, 68 °C for 30 s, 72 °C for 3.5 min and a final extension at 72 °C for 10 min. All PCR products were cloned into pGEM-T easy vector (Promega, Madison, WI, USA) and sequenced.

### 3.2. Bioinformatics Analysis of Zebrafish E-Selectin 

The nucleotide and amino acid sequences, exon–intron organization and chromosomal location as well as conserved domain were analyzed by use of the online NCBI Blast server 2.0, the zebrafish whole-genome sequence project database [33], and the Conserved Domains Database [34]. The deduced signal peptide was identified by use of SignalP (http://genome.cbs.dtu.dk/services/SignalP). We analyzed homology between the amino acid sequences of zebrafish E-selectin and other known E-selectin species by using ClustalW (http://www.ebi.ac.uk/clustalw). The phylogenetic tree was constructed by use of Mega3 and the neighbor-joining method. The genetic distance was calculated by the ρ-distance method.

### 3.3. LPS Treatment 

At 96 hpf, larvae were treated with 75 μg/L LPS for 1, 6, 10, 22, 48 and 72 h; the untreated group (0 h) was a control.

### 3.4. Real-Time Quantitative RT-PCR 

Total RNA was extracted from embryos at 0.2, 24, 48, 72 hpf and from larvae with and without LPS treatment by using TRIzol reagent (Invitrogen, Carlsbad, CA, USA) in accordance with the manufacturer’s instructions and treated with DNase I (Takara, Dalian, China). The concentration of each RNA sample was measured with a NanoDrop ND-1000 spectrophotometer (NanoDrop Technologies, Wilmington, DE, USA). Only RNA samples with an A260/A280 ratio (indication of protein contamination) of 1.9–2.1 and an A260/A230 ratio (indication of reagent contamination) > 2.0 were used for the analysis. The integrity of RNA samples was assessed by agarose gel electrophoresis. Reverse transcription of RNA into cDNA involved use of Superscript II reverse transcriptase (Invitrogen, Carlsbad, CA, USA) according to the manufacturer’s instructions. 

Real-time quantitative PCR was performed with the Bio-Rad CFX384 Real-Time PCR Detection System (Bio-Rad Laboratories, Hercules, CA, USA). Amplification was detected by using iTaq SYBR Green Supermix (Bio-Rad Laboratories). Primers for real-time quantitative PCR, with the first-strand cDNA used as a template, are in Table 1. The protocol was initial denaturation at 95 °C for 5 s, then 95 °C for 5 s, 60 °C for 30 s (fluorescent data were acquired), repeated for 40 cycles. After amplification, melting curve analysis was used to verify the authenticity of the PCR products in accordance with the manufacturer’s instructions. The target gene and β-actin were amplified in separate tubes. All reactions were performed in triplicate. Changes in the expression of target genes were calculated by the 2^−ΔΔ*C*t^ method, ΔΔ*C*t = (*C*_t,target_ − *C*_t,β-actin_)sample − (*C*_t,target_ − *C*_t,β-actin_)control [35]. The expression at 0.2 hpf was used to calibrate temporal expression, and the expression for the control was used to calibrate expression induced by LPS.

**Table 1:** Primers for real-time quantitative PCR.

Gene (Acession Number)	Prime Sequences (5′–3′)	Product Size
β-actin (AF057040)	Forward: ATGGATGAGGAAATCGCTG	130
	Reverse: ATGCCAACCATCACTCCCTG	
E-selectin (KC488324)	Forward: GCAGCACTACACCGACTTGG	196
	Reverse: TCCTTTGTTATTGGGCTCCTT	
TNF-α (AY427649)	Forward: GCTGGATCTTCAAAGTCGGGTGTA	138
	Reverse: TGTGAGTCTCAGCACACTTCCATC	
IL-1β (AY340959)	Forward: TGGACTTCGCAGCACAAAATG	124
	Reverse: GTTCACTTCACGCTCTTGGATG	

### 3.5. Whole-Mount in Situ Hybridization 

A 540-bp antisense RNA probe was generated from the zebrafish E-selectin coding sequence (245–784 bp of the cDNA sequence, primer sequences 5′-GGCAGCACTACACCGACTTG-3′ and 5′-CCTTCAGTTCATAACCCTCCTCAC-3′). The digoxigenin (DIG)-labeled antisense RNA probe was synthesized by linearising the E-selectin-T easy plasmid with NcoI and transcribing with Sp6 RNA polymerase. Whole-mount *in situ* hybridization was performed as described [36]. Briefly, embryos were fixed in 4% paraformaldehyde (Sigma-Aldrich, Saint Louis, MO, USA) at 4 °C overnight, then dehydrated with alcohol. After treatment with proteinase K, embryos were pre-hybridized and incubated for 2–5 h at 65 °C, then hybridized with probe concentration of 1 ng/µL overnight at 65 °C. After washing, samples were incubated with an alkaline-phosphatase-conjugated antibody (Roche Diagnostics, Indianapolis, IN, USA) at dilution 1:4000. Nitroblue tetrazolium/5-bromo-4-chloro-3-indolyl phosphate (NBT/BCIP, Roche) was used as the enzymatic substrate.

### 3.6. Statistical Analysis 

Statistical analysis was performed using of SPSS v12.0 (SPSS Inc., Chicago, IL, USA). Data are presented as mean ± S.D. Comparisons between groups involved one-way ANOVA. Differences were considered statistically significant at *p* < 0.05. 

## 4. Conclusion

In summary, we have successfully cloned the zebrafish E-selectin gene, which is involved in embryonic development and critical for hematopoiesis and angiogenesis and likely has a similar biological function as mammalian E-selectins. Our study may provide new ideas and information for further research and treatment of many types of inflammatory diseases including atherosclerosis, diabetes, dermatitis, asthma and cancer.

## Abbreviations

DIG, digoxigenin; ECs, endothelial cells; EGF, epidermal growth factor; hpf, hours post fertilization; IL-4, interleukin-4; IL-1β, interleukin-1β; LPS, lipopolysaccharide; NBT/BCIP, nitroblue tetrazolium/5-bromo-4-chloro-3-indolyl phosphate; RACE, rapid amplification of cDNA ends; SCR, short consensus repeat; sLe^A^, sialyl Lewis A; sLe^X^, sialyl Lewis X; TNF-α, tumor necrosis factor-α; UTRs, untranslated regions.
